# Self-Controlled Feedback Facilitates Motor Learning in Both High and Low Activity Individuals

**DOI:** 10.3389/fpsyg.2012.00323

**Published:** 2012-08-31

**Authors:** Jeffrey T. Fairbrother, David D. Laughlin, Timothy V. Nguyen

**Affiliations:** ^1^Department of Kinesiology, Recreation, and Sport Studies, University of TennesseeKnoxville, TN, USA; ^2^Department of Sport Science and Physical Education, Huntingdon CollegeMontgomery, AL, USA; ^3^DaVita Inc.Denver, CO, USA

**Keywords:** self-control, feedback, motor learning, knowledge of results, self-regulation, physical activity

## Abstract

The purpose of this study was to determine if high and low activity individuals differed in terms of the effects of self-controlled feedback on the performance and learning of a movement skill. The task consisted of a blindfolded beanbag toss using the non-preferred arm. Participants were pre-screened according to their physical activity level using the International Physical Activity Questionnaire. An equal number of high activity (HA) and low activity (LA) participants were assigned to self-control (SC) and yoked (YK) feedback conditions, creating four groups: Self-Control-High Activity; Self-Control-Low Activity; Yoked-High Activity; and Yoked-Low Activity. SC condition participants were provided feedback whenever they requested it, while YK condition participants received feedback according to a schedule created by their SC counterpart. Results indicated that the SC condition was more accurate than the YK condition during acquisition and transfer phases, and the HA condition was more accurate than the LA condition during all phases of the experiment. A post-training questionnaire indicated that participants in the SC condition asked for feedback mostly after what they perceived to be “good” trials; those in the YK condition indicated that they would have preferred to receive feedback after “good” trials. This study provided further support for the advantages of self-controlled feedback when learning motor skills, additionally showing benefits for both active and less active individuals. The results suggested that the provision of self-controlled feedback to less active learners may be a potential avenue to teaching motor skills necessary to engage in greater amounts of physical activity.

## Introduction

Recent research has indicated that giving learners control over some aspect of an instructional protocol facilitates motor learning when compared to protocols that are completely prescribed by the researcher (for a review, see Wulf, 2007). For example, the provision of self-control (SC) during practice has been shown to enhance learning in studies investigating the effects of physical guidance, video demonstrations, augmented feedback, and task scheduling on a variety of motor skills involving balance, object projection, and sequential timing (Janelle et al., 1995, 1997; Wulf and Toole, 1999; Wulf et al., 2001, 2005; Chen et al., 2002; Chiviacowsky and Wulf, 2002, 2005, 2007; Chiviacowsky et al., 2008; Wu and Magill, 2011). Even allowing participants to control the total number of practice trials they complete has been shown to enhance learning (Post et al., 2011).

A number of studies have implemented post-training questionnaires to determine why participants in SC conditions either asked for assistance or refrained from doing so. Chiviacowsky and Wulf (2002) reported that participants’ preference for feedback was linked to their perceived success on a trial. Participants in the SC condition indicated that they requested feedback after so-called “good” trials (i.e., those trials that they perceived to be successful). Participants in the yoked (YK) condition also indicated they would have preferred to receive feedback after “good” trials. These findings imply that SC participants can accurately judge the success of a trial and use this capability to self-select feedback when it was most useful – after “good” trials. Chiviacowsky and Wulf (2005) tested this idea by examining the performance and learning of a sequential timing task under two SC conditions. In one condition, participants requested feedback before a trial (self-before) and in another they requested it after a trial (self-after). If the effectiveness of previous SC manipulations depended upon the learner’s assessment of success on a trial, it would be expected that requesting feedback before a trial would not be as effective as requesting it after a trial. The results indicated that self-after condition was indeed more accurate than the self-before condition during a transfer test. Chiviacowsky and Wulf (2007) then provided additional support for the benefit of receiving feedback after “good” trials by demonstrating enhanced learning when knowledge of results (KR) was provided for the most accurate trials in a block compared to when it was provided for the least accurate trials.

Another interesting aspect of SC research is that participants in SC conditions often ask for assistance far less frequently than might be expected. Janelle et al. (1995, 1997) found that participants asked for feedback after less than 12% of trials during acquisition. Wrisberg and Pein (2002) found that participants asked to see video demonstrations before only 9.8% of trials and that 82% of these requests occurred early during the first of 3 days of practice. In addition, 92% of the requests during the second and third days occurred during the first three trials (out of 31 total). Additionally, Wulf and Toole (1999) reported that participants requested physical guidance 92% of the time on the first trial, but only 25% of the time on the last trial of practice.

Several explanations have been forwarded to account for how SC manipulations work in motor learning. Janelle et al. (1995, 1997) argued that SC fosters deeper information processing. Others have suggested that SC may enhance participant motivation (McNevin et al., 2000). Based on the evidence that participants reported asking for feedback after “good” trials, Chiviacowsky and Wulf (2002) claimed that SC feedback conditions allow learners to adopt a learning strategy based on the successful estimation of their own errors. In addition, they claimed that the requested feedback is used to confirm the participant’s success rather than correct errors. The relatively low number of requests for instructional assistance seen in several studies (e.g., Wrisberg and Pein, 2002) also suggests that learners understand when they need instructional support and that they should decrease their requests for assistance as they gain proficiency. Furthermore, Chiviacowsky and Wulf (2002, 2005) argued that SC allows participants to tailor the instructional experience to match their individual needs and preferences for self-regulation.

These explanations point to the important role that individual differences may play in SC feedback protocols. One such individual difference that might influence how participants utilize the provision of SC may be the varying degrees of their experience with movement skills. Individuals who engage in relatively low levels of physical activity will typically have fewer opportunities to develop and strengthen motor skills compared to their more active peers. Low levels of motor competence can, in turn, serve as a barrier to future engagement in physical activity. This perspective is consistent with arguments by Stodden et al. (2008) that a failure to adequately acquire fundamental motor skills during childhood or adolescence will preclude the desired range of physical activity as an adult. There is evidence that low fundamental motor skill proficiency in childhood is linked to low physical activity and fitness during adolescence (Stodden et al., 2009). Additionally, only a small number of adolescents in the US engage in desirable amounts of physical activity and few of those who do actually maintain such behaviors into young adulthood (Gordon-Larsen et al., 2004; Barnett et al., 2008; Barnett, 2009; Lubans et al., 2010). So, it is logical that the link between motor skill proficiency and physical activity would persist into young adulthood. Indeed, the Centers for Disease Control and Prevention acknowledge *lack of skill* as among the more common barriers to physical activity (Centers for Disease Control and Prevention, 2011; Epping et al., 2012). Because self-controlled feedback has been repeatedly shown to facilitate motor skill learning, its potential as an effective method to help inactive people overcome *skill barriers* to physical activity merits consideration. As used in this paper, the term *skill barrier* refers to a situation in which a relatively low level of proficiency in completing certain motor skills serves as an obstacle to increasing physical activity through participation in a skill-based movement. For example, a person who lacks basic competency in overhand throwing will face a skill barrier to participating in recreational softball. The purpose of this study, therefore, was to examine the effects of self-controlled feedback on motor learning for adults who report engaging in different levels of physical activity.

There are some reasons to expect that individuals who engage in relatively low levels of physical activity might behave differently compared to more active peers in a self-controlled feedback protocol. Schmidt (1975) argued that connecting sensory consequences with the outcome of a movement is an important aspect of motor learning and prominent explanations for SC benefits have emphasized the role of such self-evaluation of performance (e.g., Chiviacowsky and Wulf, 2002, 2005). It may be that physically inactive individuals will have difficulty interpreting sensory consequences when learning certain types of skills given their relative inexperience engaging in fundamental motor skills. There is some evidence for this in older adults (Buatois et al., 2007) and if also true for adults in general, we might expect diminished effectiveness of self-controlled feedback compared to more active counterparts or perhaps at least some different behaviors related to how SC is exercised. Presumably, it is likely that active individuals by virtue of more experience with movement skills will outperform their less active counterparts. It is possible that such a difference in performance might prompt different patterns of feedback requests. For example, relatively poor performance might lead less active individuals to seek corrective feedback (after “bad” trials) more often than active individuals. Whether or not divergent patterns of feedback requests would influence the effectiveness of SC feedback is unknown, but identifying such behavior would be helpful to inform the expectations of practitioners implementing SC protocols and to further current thought on how feedback is used in motor learning (Chiviacowsky and Wulf, 2007). Of course, it is also possible that SC feedback will prompt similar behavior from both active and less active individuals, and will also facilitate motor learning for both groups. Such a demonstration would be important in establishing SC protocols as potentially effective interventions in helping less active people who struggle with skill barriers to physical activity participation.

## Materials and Methods

### Participants

Participants were 48 college-age volunteers (24 male; 24 female). The average age of participants was 21.31 years (SD = 2.73 years). All but four were right handed. Prior to the experiment, all participants provided informed consent. None of the participants had any prior experience with the experimental task or procedures. Participants were screened to exclude those with past experience in sports and activities that require the projection of an implement using the upper extremities (e.g., baseball, shot put, or javelin).

### Apparatus and task

The task was similar to the one used by Chiviacowsky and Wulf (2007), but with the scoring scale reversed. It required a blindfolded participant to toss a beanbag (100 g) with the non-preferred arm at the center of a circular target placed on the floor. The radius of the target was 10 cm and it was surrounded by nine additional circles which each increased the radius by 10 cm. The participant stood behind a line located 3 m from the center of the target. Each toss was scored using a point system in which zero points were awarded for landing on the target (i.e., the 10-cm circle). The score was increased by 10 points for each successive circle moving away from the center of the target, such that a lower score indicated less error.

### Procedure

Upon arriving at the laboratory, participants were assigned to High Activity (HA) and Low Activity (LA) groups according to an initial screening of their level of physical activity using the energy expenditure estimates based on the International Physical Activity Questionnaire (IPAQ; Craig et al., 2003). The criteria for activity level classifications were as follows. Participants were assigned to the HA condition if their IPAQ responses led to an estimated total energy expenditure of at least 1600 metabolic equivalent-minutes/week (MET-min). Participants with totals less than 1600 MET-min/week were assigned to the LA condition. These criteria resulted in 24 participants each in the HA condition (IPAQ estimated energy expenditure: *M *= 5,063 MET-min/week; SD = 3300 MET-min/week), and LA condition (IPAQ estimated energy expenditure: *M* = 270 MET-min/week; SD = 259 MET-min/week). Participants in each of these conditions were then randomly assigned to either a SC feedback condition or a YK condition to create four groups [Self-Control-High Activity (SC-HA); Self-Control-Low Activity (SC-LA); Yoked-High Activity (YK-HA); and Yoked-Low Activity (YK-LA)]. Participants were yoked man-to-man, woman-to-woman, HA-to-HA, and LA-to-LA.

Prior to the acquisition phase, each participant was given written instructions that were also read aloud by the experimenter and then allowed to complete three practice trials to become familiar with the experimental procedures. During the acquisition phase, participants completed 60 total trials in 10 blocks. All participants were instructed to toss the beanbag as close to the center of the target as possible. Participants in the SC condition were told that they could ask for feedback after any trial, and that feedback would not be provided unless they requested it. Participants in the YK condition were told that they would receive feedback after some trials but not others. YK condition feedback schedules matched the schedules created by their counterparts in the SC condition. When feedback was administered, it was provided verbally in the form of KR on the error score for the trial and the direction in which the beanbag was located with respect to the center of the target (e.g., “80, long, left,” “30, short, right,” or “40, long”). For each trial, the experimenter recorded the score for the toss and whether or not KR was administered. The intertrial interval was regulated with a chronometer and was approximately 10 s. At the end of acquisition, all participants completed a post-training questionnaire regarding the feedback they received. The post-training questionnaire was adapted from Chiviacowsky and Wulf (2002). The questionnaire asked SC participants when they requested feedback (e.g., after mostly good trials or after mostly bad trials) and when they did *not* ask for feedback. The YK participants were asked if they felt they received feedback when it was most needed. If they indicated this was the case, they were asked when they thought they received it (e.g., mostly after good trials or mostly after bad trials). If they indicated they did not receive feedback when it was most needed, participants were asked when they would have *preferred* to receive feedback.

After approximately 24 h, participants returned to the lab to take the retention and transfer tests. The retention test consisted of 12 trials administered in two blocks using the same task and procedures as in acquisition with the exception that KR was not provided. After a 10-min break, the transfer test was administered. The transfer test was identical to the retention test except the distance from which the participants tossed was increased to 5 m to assess the extent to which participants were capable of adapting to a novel but related task demand. During all three experimental phases, participants were allowed to remove the blindfold and view the empty target after the completion of each block of trials.

### Data treatment and analysis

Error scores were recorded for each trial during acquisition, retention, and transfer. Scores were determined by the assigned point value for the circle in which the beanbag landed. When a beanbag came to rest on two scoring rings, it was awarded the score corresponding to the ring in which the center of the beanbag rested. Each beanbag had a small mark on its center to facilitate this judgment. When the center of the beanbag was located on a line, it was given the lower score. A toss was awarded zero points if it landed on the 10-cm radius target. The score increased by 10 points for each successive circle moving away from the target (by 10-cm increments). Tosses not landing within any of the circles were awarded a score of 100 points. For each participant in the SC condition, the trials after which feedback was received were also recorded and the frequency of feedback requests was calculated for each trial block. For the post-training questionnaire, the number of responses to each question was tabulated for the SC and YK conditions.

For acquisition, error scores were analyzed using a 2 (feedback condition: SC vs. YK) × 2 (activity level: HA vs. LA) × 10 (trial block) analysis of variance with repeated measures of the last factor. Frequency of feedback requests in the SC condition was analyzed using a 2 (activity level: HA vs. LA) × 10 (block) analysis of variance with repeated measures on the last factor. To examine whether or not participants in the SC condition requested feedback after “good” trials, the error scores for feedback-trials and no-feedback-trials were analyzed using a 2 (activity level: HA vs. LA) × 2 (trial type: feedback vs. no-feedback) × 2 (half of acquisition trials: first vs. second) analysis of variance with repeated measures on the last two factors. For retention and transfer, error scores were analyzed using separate 2 (feedback condition: SC vs. YK) × 2 (activity level: HA vs. LA) × 2 (block) analyses of variance with repeated measures on the last factor. When appropriate, *F*-ratios involving repeated measures factors were reported with the Greenhouse–Geisser *df* adjustment. Partial eta-squared values (η^2^) were reported to indicate effect sizes for significant results. Follow-up testing was conducted using Sidak *post hoc* procedures. For all analyses, alpha was set at 0.05. Frequency of responses from the post-training questionnaire were tabulated and reported descriptively.

## Results

### Acquisition

**Figure 1:**
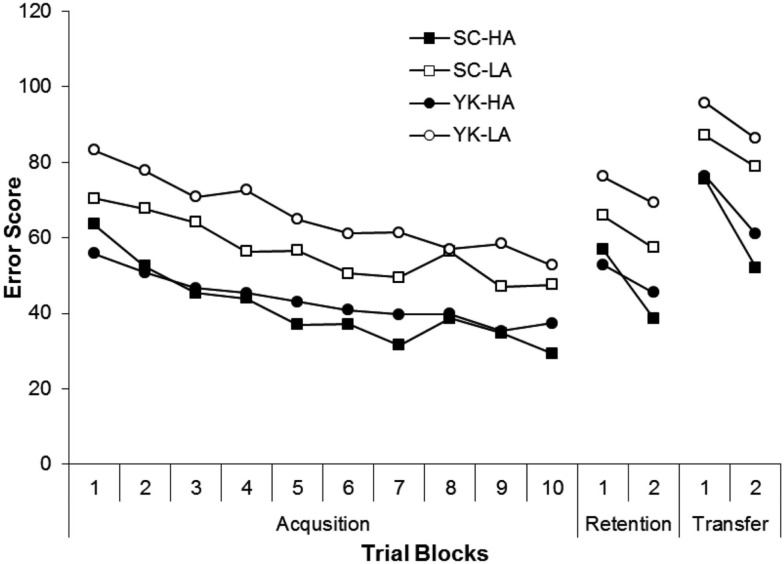
**Mean error scores for the Self-Control-High Activity (SC-HA), Yoked-High Activity (YK-HA), Self-Control-Low Activity (SC-LA), and Yoked-Low Activity (YK-LA) groups during each trial block of acquisition, retention, and transfer**. A lower score represents more accurate performance.

Figure 1 shows mean error scores for each condition during acquisition. The SC and HA conditions performed more accurately (i.e., produced lower scores) than the YK and LA conditions, respectively. Both conditions showed increased accuracy across trial blocks. For the HA condition, the provision of SC did not appear to have an effect on performance whereas it appeared to facilitate performance for the LA condition. These observations were supported by the significant main effects for feedback condition, *F* (1, 44) = 6.26, *p* = 0.016, η^2^ = 0.13, activity level, *F* (1, 44) = 67.80, *p* < 0.001, η^2^ = 0.61, and block, *F* (9, 396) = 21.07, *p* < 0.001, η^2^ = 0.32. The interactions between feedback condition and block, *F* (9, 396) = 0.73, *p* = 0.732, feedback condition and activity level, *F* (1, 44) = 2.49, *p* = 0.122, activity level and block, *F* (9, 396) = 0.26, *p* = 0.953, and feedback condition, activity level, and block, *F* (9, 396) = 0.95, *p* = 0.456, were not significant.

**Figure 2:**
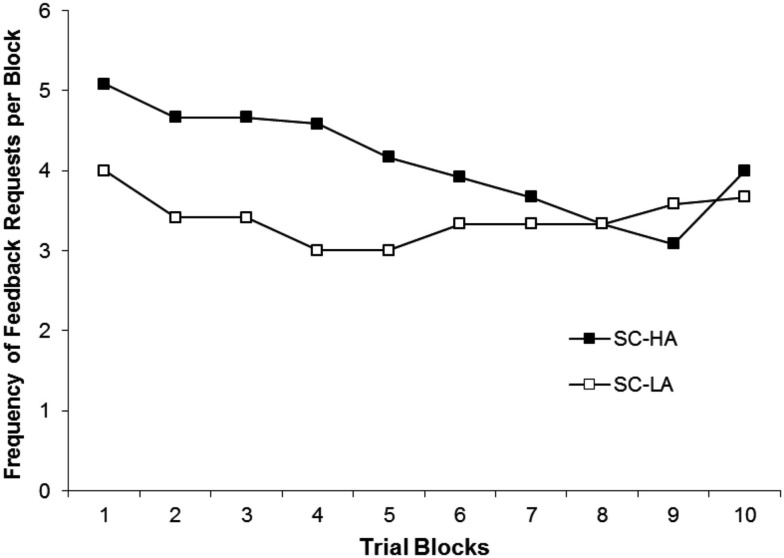
**Mean frequency of feedback requests for the Self-Control-High Activity (SC-HA) and Self-Control-Low Activity (SC-LA) groups during each trial block of acquisition**.

Figure 2 shows mean frequency of feedback requests for the SC condition during acquisition. The SC-HA condition requested feedback more frequently than the SC-LA condition during the initial blocks of acquisition. However, the frequency of feedback requests was similar by the end of acquisition. This was due to a relatively high frequency of requests by the SC-HA condition during Blocks 1–5. The frequency of requests by the SC-LA condition remained relatively stable throughout acquisition. These observations were supported by the significant main effects for block, *F* (9, 198) = 3.31, *p* = 0.009, η^2^ = 0.31, and activity level, *F* (1, 22) = 4.50, *p* = 0.036, η^2^ = 0.19, and the significant interaction between activity level and block, *F* (9,198) = 2.61, *p* = 0.031, η^2^ = 0.11. *Post hoc* procedures following the Activity Level × Block interaction revealed that the frequency of feedback requests was significantly higher for the SC-HA condition compared to the SC-LA condition during each of the first five trial blocks (*p* < 0.048 for all comparisons).

**Figure 3:**
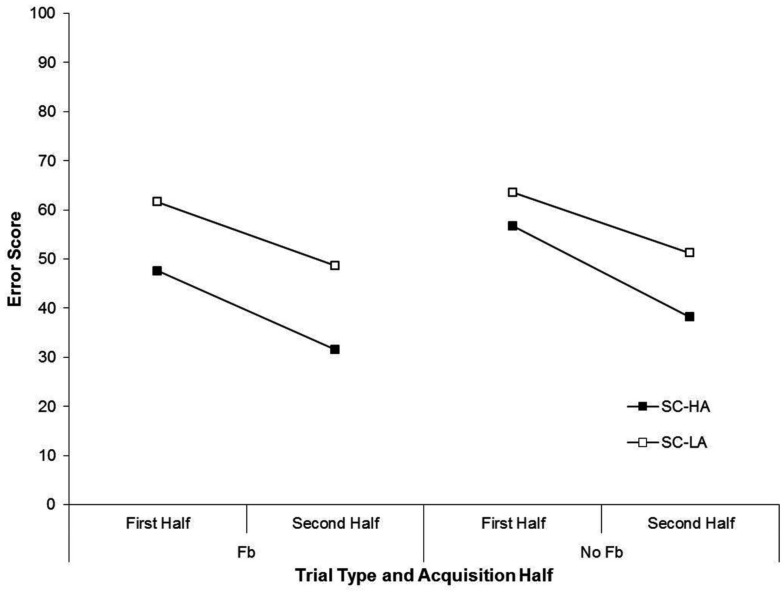
**Mean error scores for the Self-Control-High Activity (SC-HA) and Self-Control-Low Activity (SC-LA) groups as a function of trial type (feedback – Fb vs. no-feedback – No Fb) and acquisition half**. A lower score represents more accurate performance.

Figure 3 shows mean error scores for feedback-trials and no-feedback-trials for the SC condition during the first and second halves of acquisition. Feedback-trials were more accurate than no-feedback-trials. The HA condition was more accurate than the LA condition, with both conditions improving from the first to second halves. These observations were supported by the significant main effects for trial type, *F* (1, 20) = 9.31, *p* = 0.006, η^2^ = 0.32, activity level, *F* (1, 20) = 11.91, *p* = 0.003, η^2^ = 0.37, and half, *F* (1, 20) = 22.90, *p* = 0.001, η^2^ = 0.53. The interactions involving trial type and activity level, *F* (1, 20) = 2.70, *p* = 0.116, trial type and half, *F* (1, 20) = 0.01, *p* = 0.914, activity level and half, *F* (1, 20) = 0.66, *p* = 0.427, and trial type, activity level, and half, *F* (1, 20) = 0.08, *p* = 0.785, were not significant.

### Retention

Figure 1 shows mean error scores for each condition during retention and transfer. During retention, the SC and YK conditions both reduced their error scores from the first block to the second. In addition, the HA condition was more accurate than the LA condition. These observations were supported by the significant main effects for block, *F* (1, 44) = 11.60, *p* < 0.001, η^2^ = 0.21, and activity level, *F* (1, 44) = 18.84, *p* < 0.001, η^2^ = 0.30. The main effects for feedback condition, *F* (1, 44) = 2.12, *p* = 0.152, η^2^ = 0.30, and the interactions between feedback condition and block, *F* (1, 44) 1.09, *p* = 0.302, η^2^ = 0.02, activity level and block, *F* (1, 44) = 0.70, *p* = 0.407, η^2^ = 0.02, and feedback condition, activity level, and block, *F* (1, 44) = 0.59, *p* = 0.447, η^2^ = 0.02, were not significant.

### Transfer

During transfer, the SC and HA conditions were more accurate than the YK and LA conditions, respectively. In addition, all conditions improved across blocks. This improvement was more pronounced for the HA condition than for the LA condition. These observations were supported by the significant main effects for feedback condition, *F* (1, 44) = 4.98, *p* = 0.031, η^2^ = 0.10, activity level, *F* (1, 44) = 51.14, *p* < 0.001, η^2^ = 0.54, and block, *F* (1, 44) = 60.39, *p* < 0.001, η^2^ = 0.58, and the significant interaction between activity level and block, *F* (1, 44) = 8.53, *p* = 0.005, η^2^ = 0.16. *Post hoc* procedures following the Activity Level × Block interaction indicated that the HA condition was more accurate than the LA condition during both blocks (*p* < 0.001). The interactions between feedback condition and block, *F* (1, 44) = 0.88, *p* = 0.355, η^2^ = 0.02, feedback condition and activity level, *F* (1, 44) = 0.32, p = 0.578, η^2^ = 0.54, and feedback condition, activity level, and block, *F* (1, 44) = 1.64, *p* = 0.207, η^2^ = 0.04, were not significant.

### Questionnaire results

Table 1 shows the number of responses indicating when participants either requested or would have preferred to receive feedback. For the SC condition, the majority of participants indicated that they requested feedback mostly after what they believed to be a “good” trial (*n *= 14) while none indicated asking for feedback after “bad” trials. Other responses indicating when SC participants requested feedback included equally after “good” and “bad” trials (*n *= 3), randomly (*n* = 5), and some other criteria (*n* = 2). Both of the participants who indicated the *other* criteria noted that they requested feedback to seek information about the outcome of the toss. They did not comment on whether the desire for this information was related to their performance on the previous trial. The majority of SC condition participants also indicated that they did *not* request feedback after what they believed to be a “bad” trial (*n *= 18). No participant indicated *not* asking for feedback following good trials. The *other* category for *not* asking was indicated by five SC-HA participants and one SC-LA participant. Four of the SC-HA participants provided additional information after responding with the *other* category. One indicated having no specific reason, but simply wanting to learn the task, one indicated doing so randomly, and two indicated that they did not ask for feedback after “good” trials. Adding these two latter responses to the tabulation of previous categories raised the total number of participants who indicated *not* asking for feedback after “good” trials from zero to two. There were no clear differences between SC-HA and SC-LA participants in terms of questionnaire responses. For both groups, most participants asked for feedback after “good” trials and did *not* ask for feedback after “bad” trials.

**Table 1:** **Number of responses for the Self-Control-High Activity (SC-HA), Self-Control-Low Activity (SC-LA), Yoked-High Activity (YK-HA), and Yoked-Low Activity (YK-LA) groups indicating when they asked for (SC) or would have preferred (YK) to receive feedback**.

Feedback condition	Activity level	Types of trials^a^			
		Good	Bad	Other^b^	Total
SC	HA	6	0	6	12
	LA	8	0	4	12
YK	HA	5	0	6	11^c^
	LA	7	3	2	12

*SC, Self-Control; YK, Yoked; HA, High Activity; LA, Low Activity*.

*^a^Indicates after which type of trial participants indicated they either asked for or would have preferred to receive feedback*.

*^b^Other includes responses indicating after both good and bad trials equally, randomly, or some other reason for both the SC and YK conditions and also that when feedback was delivered did not matter for the YK condition*.

*^c^One participant in the YK-HA group did not complete the post-training questionnaire, so reported frequencies are for the other 23 participants in the YK condition*.

For the YK condition, the majority of the participants indicated that they felt they did not receive feedback when they needed it most (*n* = 13) while a smaller number (*n *= 10) indicated they did. More YK participants indicated they would to have preferred feedback after what they believed to be a “good” trial (*n *= 12) than those that indicated a preference for feedback following a “bad” trial (*n *= 3). Other responses for the YK participants who felt they received feedback when needed included equally after “good” and “bad” trials (*n* = 3) and randomly (*n* = 4). One of the YK participants who did not receive feedback when needed indicated the *other* category and noted a preference for receiving feedback equally within each block of trials (e.g., three trials in each block). As with the SC participants, there were no clear differences between the responses for the YK-HA and YK-LA groups and, overall, most YK participants indicated a preference for feedback after “good” trials.

## Discussion

The purpose of the current study was to examine the effects of self-controlled feedback on the learning of a simple movement skill by individuals of differing levels of physical activity. Presumably, individual differences in capabilities related to movement skills might vary with those related to activity level (e.g., Stodden et al., 2008, 2009), in part because individuals engaging in low levels of physical activity simply experience fewer opportunities to learn movement skills compared to their active counterparts. Thus, it is likely that individuals who are relatively inactive will face a *skill barrier* when attempting to engage in physical activity (Centers for Disease Control and Prevention, 2011). Self-controlled feedback benefits have been shown for learning a variety of motor skills (e.g., Janelle et al., 1995, 1997; Chen et al., 2002; Chiviacowsky and Wulf, 2002), but are thought to operate in part through mechanisms related to individual differences (Chiviacowsky and Wulf, 2002). Thus, there was a need to examine the effectiveness of self-controlled feedback for both low and high activity individuals to determine if it might be a profitable intervention in addressing *skill barriers*.

The most important finding from the current study was that SC of KR conferred an equivalent learning benefit (as seen in Transfer) for both the HA and LA conditions. This demonstration provides the first step in establishing SC protocols as effective interventions in teaching less active individuals the motor skills they need to overcome skill barriers to healthful physical activity. Although the LA groups did perform at a lower proficiency level than the HA groups throughout the study, the benefits of SC were seen in both activity level groups with no qualifying interaction. Previous research has demonstrated the generalizability of SC effects across different types of tasks (e.g., basketball, non-dominant arm throwing, and key-pressing; Janelle et al., 1997; Chen et al., 2002; Wulf et al., 2005), types of instructional support (e.g., feedback, physical guidance, and amount of practice; Wulf and Toole, 1999; Chiviacowsky and Wulf, 2002; Post et al., 2011), and age (e.g., Chiviacowsky et al., 2008). The current results were the first to our knowledge to demonstrate that SC effects can also generalize across individual differences in young adults (e.g., activity levels). This finding is of particular importance as relatively low levels of motor proficiency are increasingly viewed as a potential barrier to participation in physical activity interventions designed to prevent or reverse obesity in inactive populations. The current results indicate that the provision of SC is an effective mode of instruction to support efforts to reduce such barriers to physical activity.

The lower proficiency level in the LA groups raised another potential issue important in efforts to better understand SC effects. Specifically, we speculated in the introduction that different levels of performance might prompt different patterns of feedback requests by less active individuals or different reasons for requesting feedback. The results showed that for feedback requests this was the case, with the LA participants making significantly fewer feedback requests during the first half of acquisition. In addition, the reduction of feedback requests seen in previous research (e.g., Wulf and Toole, 1999) was only observed for the HA participants. The SC-LA group maintained a fairly stable average number of requests per block. Importantly, however, the reasons for requesting feedback did not differ between the SC-LA and SC-HA groups, with both indicating they more frequently asked for feedback following good trials than bad trials. In addition, the analyses comparing good (feedback) and bad (no-feedback) trials supported the idea that participants in both groups could effectively self-evaluate their performance. Thus, the lower proficiency levels seen in the SC-LA group did not seem to trigger a greater need for corrective feedback following bad trials compared to the SC-HA group. The lower frequency of feedback requests by the SC-LA during the first half of acquisition was an interesting finding. Based on ideas about stages of learning (e.g., Fitts and Posner, 1967), it might be expected that learners classified with LA levels would be more likely to request feedback *more* frequently. The observed pattern may simply reflect the relative performance levels of the SC-LA and SC-HA groups. Both preferred feedback after “good” trials, but the SC-LA group had fewer “good” trials. Unfortunately, the data from the current experiment cannot fully address this issue and future studies should perhaps be directed toward understanding participants’ operational definitions of so-called “good” trials. This dilemma does not, however, undermine the importance of the current demonstration that the less active participants *reported* asking for feedback after “good” trials, were able to identify these trials, and ultimately benefited from self-controlled feedback. Thus, the current results support the idea that self-controlled feedback effects operate similarly in individuals of varying levels of physical activity. The findings also indicate that practitioners implementing self-controlled feedback protocols with less active individuals might expect lower levels of feedback requests during the early parts of practice and, more generally, that they should be aware of tendencies to provide undesired corrective feedback when not needed.

Together, the current results were consistent with Chiviacowsky and Wulf’s (2002) argument that self-evaluation plays an important role in SC effects and provided no support for the notion that a less active lifestyle leads to deficits in processing sensory information and evaluating movement performances for young adults learning a relatively simple task. These findings offer a potential direction for research addressing physical activity in inactive populations. Despite limited movement experiences, the less active individuals were able to interpret the sensory consequences of their movements, choose feedback after “good” trials, and learn more effectively when given control over feedback presentation. Thus, it appears that allowing less active individuals to control some aspect of a learning environment can lead to more effective learning. In many cases, the initial phase of a physical activity program involves learning simple movement skills needed to complete exercises. If providing SC enhances such learning, it may be a valuable method of assisting less active individuals as they attempt to become more physically active.

Although the current results showed a learning benefit during transfer, there were no differences due to SC during retention. This pattern is not unprecedented in the SC literature or in motor learning research in general (e.g., Wulf and Lee, 1993; Wrisberg and Wulf, 1997; Lai and Shea, 1998; Chiviacowsky and Wulf, 2002). As Chiviacowsky and Wulf (2002) noted, it may be that transfer is a more sensitive measure of learning than retention. Figure 1 illustrates that both the SC and YK conditions increased their mean error scores when moving to the transfer test from retention. Interestingly, the pattern of results (as indicated by the respective means of each group) was similar to that seen during retention, supporting the idea of transfer as a more sensitive measure than retention. The primary novel demand introduced by the transfer test was the requirement to toss the beanbag more forcefully to compensate for the longer distance to the target. Because SC benefits are thought to be related to self-evaluation, it may be that engaging in the process of selecting feedback-trials strengthens the learner’s capability to scale force more effectively when faced with a novel demand. Although both the SC and YK conditions were provided with objective information about performance when feedback was provided, only the SC condition could choose to receive this information after what they perceived to be a good trial. It may be that SC participants benefited because they chose to receive feedback for trials that fell within a more meaningful range of error scores. The fact that error scores did vary from trial to trial, even for the best performers, illustrated that participants engaged in scaling their tossing forces. If SC of feedback strengthened the participants’ understanding of how to more effectively scale tossing forces, it is plausible that they would have an advantage when faced with a transfer demand requiring such scaling.

Another interesting finding from the present study related to the initial differences in performance between the two activity level groups, which persisted throughout all three experimental phases. The lower proficiency of the LA group in performing the motor skill used in this study is consistent with ideas about so-called *skill barriers* (Stodden et al., 2008, 2009; Centers for Disease Control and Prevention, 2011; Epping et al., 2012). This finding also raises important questions related to the underlying cause(s) of the decrement and how long it might be expected to last. The most straightforward explanation is that less active individuals simply lack experience in controlling their movements and acquiring more experience will remedy skill deficits. Although it is well established that effective practice on any given task will eventually lead to performance improvements, the issue raised by the current experiment is whether or not such experience will also increase the likelihood that performance will also be improved when introduced to a *novel* motor skill. In other words, less active individuals might be equivalent to their active peers for a task on which they have equivalent experience (e.g., video games, texting, or computer use), but might be disadvantaged when it comes to acquiring new movement skills. We recommend that future research be directed toward determining if performance deficits can be resolved with more practice. The present results suggest that the impact of an inactive lifestyle is not related to the cognitive aspects of motor performance and learning. Indeed, both activity level groups benefited from the SC manipulation which presumably operates on cognitive processes related to interpreting information. Regardless of the cause or ultimate duration of less proficient performance of the LA group, it seems prudent to recognize that activity level differences have the potential to introduce unwanted variance into samples used in short-term motor learning protocols and researchers might want to control or measure the associated effects.

It has also been noted that the provision of SC should logically enhance participant motivation (McNevin et al., 2000), an idea that fits well with the paradigm’s roots in social learning theory, self-regulation research in educational settings, and self-determination theory (Bandura, 1977; Zimmerman, 1989; Deci and Ryan, 2000). The results from previous SC studies, along with those from the current one, that have reported participants seeking feedback following good trials (Janelle et al., 1995, 1997; Wulf and Toole, 1999; Wulf et al., 2001, 2005; Chiviacowsky and Wulf, 2002, 2005, 2007; Wulf, 2007) are consistent with the idea that confirmation of success on such trials might act to enhance self-efficacy and motivation. Thus, the value of SC in teaching movement skills to physically inactive individuals could be further enhanced by the potential for positive motivational effects to enhance physical activity adherence. It is recommended that future research explore the ways in which SC influences motivation in individuals who engage in a variety of different levels of physical activity.

### Conclusions

The findings of the present study led to the following conclusions: (a) the provision of SC of feedback facilitated transfer of motor skills when compared to a YK condition regardless of activity level, (b) when provided SC, HA participants asked for feedback more frequently than LA participants, (c) when provided SC, both high and low activity participants reported asking for feedback primarily after “good” trials, and (d) LA participants performed with lower proficiency than HA participants throughout all experimental phases. To our knowledge, this is the first study to demonstrate the generalizability of SC effects across individual differences in a young adult population. The results provided initial empirical evidence that can be interpreted as supporting the notion that the provision of SC when learning new movement skills can play a positive role in overcoming one potential barrier to adult participation in physical activity (Stodden et al., 2008). The results also revealed a need for further examinations of the relationship between physical activity levels and motor learning.

## Conflict of Interest Statement

The authors declare that the research was conducted in the absence of any commercial or financial relationships that could be construed as a potential conflict of interest.
